# Tech-trends in orthopedics 2018

**DOI:** 10.1080/17453674.2018.1518806

**Published:** 2018-10-23

**Authors:** Max Gordon

**Affiliations:** Department of Clinical Sciences at Danderyd Hospital, Karolinska Institute, Stockholm, Sweden

A trend is a direction in which something is developing; in medical specialties this can be viewed as the phase before something becomes evidence-based medicine. Early adopters are those that start using a technology as soon as it becomes available, i.e., individuals that are sensitive to trends. Within the medical field, orthopedics has a long track record of being an early adopter.

Unfortunately, discriminating between positive and negative trends can be difficult; while the anterolateral approach for hip fractures (Enocson et al. 2009) has become evidence-based medicine, other trends such as resurfacing arthroplasties (Reito et al. 2017) and primary surgery for clavicle fractures (Ban et al. 2016) have failed. As increasing amount of innovation occurs in the digital space, it is important that we transfer the lessons from surgical trends to these innovations.

## Augmented reality

In this issue of *Acta Orthopaedica*, Gregory et al. (2018) show us a glimpse of how we may perform surgery a few years down the road. Their article explores the use of mixed reality (also known as augmented reality) for surgery, a technology in which a computer-generated image is superimposed on top of the visual field. This is different from its sibling, virtual reality (VR), where the user is completely immersed in the computer-generated reality. Earliest mentions of augmented reality in PubMed go back to the mid-1990s (Lavallée et al. 1995), but we have only recently gained the technology that can live up to the original visions.

While Gregory et al.’s paper shows the first stumbling steps, it feels quite plausible that this could be just as common as cordless power tools in the years to come. The field where augmented reality probably has the strongest foothold is neurosurgery (Kersten-Oertel et al. 2013), but unfortunately the evidence of whether outcomes actually improve is scarce (Meola et al. 2017). It will be interesting to see if augmented reality will become a trend—at the moment the jury is still out; it is even uncertain whether they have assembled.

## Computer-assisted surgery

Computer-assisted surgery (CAS) has, contrary to augmented reality, been both widely implemented and tested, especially for knee surgery. By mapping CT/MRI scans to the actual bone the system can navigate for the surgeon, thereby allowing improved implant positioning and smaller incisions (Dutton et al. 2008). An interesting randomized controlled trial from Petursson et al. (2017) showed no benefit in regard to RSA migration patterns, i.e. no signs of improved implant survival despite better positioning. Recently, they published patient reported outcomes from the same RCT where CAS patients were better on some subscales (Petursson et al. 2018), this should though be viewed with caution as knee function was a secondary outcome and the subscales were never mentioned in the ClinicalTrials.gov registration. Large registry cohorts have, though, not been able to clearly demonstrate the benefits (Roberts et al. 2015, Dyrhovden et al. 2016) and there are reports of falling utilization rates (Gholson et al. 2017).

Some believe that patient-specific guides will succeed CAS. By skipping the cumbersome mapping of the CT/MRI to the bone structure, you get patient specific 3D-printed saw guides that can both reduce the surgery time and improve accuracy. Unfortunately, this has also failed to translate into any tangible patient benefits (Chareancholvanich et al. 2013, Victor et al. 2014, Leeuwen et al. 2018). The trend is certainly looking grim for these types of technologies.

## Robot-assisted surgery

One interesting development is the development of robot-assisted orthopedic surgery. At the moment we are still far from fully autonomous robots, but simply assisting the human could be an efficient way of providing accuracy (Marchand et al. 2018). The long history of robotic surgery publications (Kwoh et al. 1988, Bach et al. 2002) suggests that the trend has some difficulty catching on. A quick glance at other surgical fields shows that the robot trend has certainly made a huge impact; the Da Vinci surgical system continues to grow year on year (Peters et al. 2018).

## Artificial intelligence

One of the strongest general tech-trends recently is the revival of neural networks, also known as deep learning, a form of artificial intelligence (AI). Through my work in applying AI for interpreting radiographs (Olczak et al. 2017) I am certainly biased, but I believe there is great potential in the technology. For instance, Chung et al. (2018) recently showed how it could be used for classifying humerus fractures, providing hope for solving the low classification reproducibility (Audigé et al. 2004). It is hoped it could also make the classifications more clinically relevant (Shehovych et al. 2016). There is also interesting work for classifying knee osteoarthritis (Tiulpin et al. 2018), the authors of which have released their dataset for anyone to experiment with (open data).

Artificial intelligence is, however, not limited to image interpretations. The technology is about finding structure in data; it is similar to regular statistics but does this on an entirely different scale. It is already being implemented for augmented reality (Pollefeys 2017) and can in theory enhance anything that analyzes patterns. At the same time, there are indications that clinical applications struggle to deliver (Ross and Swetlitz 2017). The struggles suggest that we still have a lot to learn and, based on my own experience, it takes time to appreciate the full complexity. For instance, an orthopedic surgeon is well aware that a fracture is not a question of yes/no, but has almost infinite subtle interpretations.

## Final thoughts

We know that predicting the next big thing is hard (Denrell and Fang 2010), but at the same time it is interesting to survey the area and think of what direction we want the future to take. There are even some who believe that we are the ones who shape the future. Most of the things mentioned in this paper will require great efforts and a great number of people; fortunately, it has never been easier to participate in this endeavor.
